# The Extent to Which Performance-Based Financing Programs' Operations Manuals Reflect Rights-Based Principles: Implications for Family Planning Services

**DOI:** 10.9745/GHSP-D-19-00007

**Published:** 2019-06-24

**Authors:** Marie S. Cole, Victoria Boydell, Karen Hardee, Ben Bellows

**Affiliations:** a PAI, Washington, DC, USA.; b Graduate Institute, Geneva, Switzerland.; c What Works Association, Morristown, NJ, USA.; d Population Council, Washington, DC, USA.

## Abstract

Recognition is growing that development programs need to be guided by rights as well as to promote, protect, and fulfill them. Drawing from a content analysis of performance-based financing (PBF) implementation manuals, we quantify the extent to which these manuals use a rights perspective to frame family planning services. PBF is an adaptable service purchasing strategy that aims to improve equity and quality of health service provision. PBF can contribute toward achieving global family planning goals and has institutional support from multiple development partners including the Global Financing Facility in support of Every Woman Every Child. A review of 23 PBF implementation manuals finds that all documents are focused largely on the implementation of quality and accountability mechanisms, but few address issues of accessibility, availability, informed choice, acceptability, and/or nondiscrimination and equity. Notably, operational inclusion of agency, autonomy, empowerment, and/or voluntarism of health care clients is absent. Based on these findings, we argue that current PBF programs incorporate some mention of rights but are not systematically aligned with a rights-based approach. If PBF programs better reflected the importance of client-centered, rights-based programming, program performance could be improved and risk of infringing rights could be reduced. Given the mixed evidence for PBF benefits and the risk of perverse incentives in earlier PBF programs that were not aligned with rights-based approaches, we argue that greater attention to the rights principles of acceptability, accessibility, availability, and quality; accountability; agency and empowerment; equity and nondiscrimination; informed choice and decision making; participation; and privacy and confidentiality would improve health service delivery and health system performance for all stakeholders with clients at the center. Based on this review, we recommend making the rights-based approach explicit in PBF; progressively operationalizing rights, drawing from local experience; validating rights-based metrics to address measurement gaps; and recognizing the economic value of aligning PBF with rights principles. Such recommendations anchor an aspirational rights agenda with a practical PBF strategy on the need and opportunity for validated metrics.

## Abstract (precis)

Rights principles should be prioritized and more clearly stated in performance-based financing (PBF) guidance and operational documents. Additional research, including development of validated measurement metrics, is needed to help PBF programs systematically align with rights-based approaches to health care including family planning.

## INTRODUCTION

Since the 1994 International Conference on Population and Development, the family planning movement has increasingly focused on person-centered or client-centered approaches to ensure individuals and couples can freely determine whether and when to bear children.^1^^–^^4^ However, significant barriers remain, inhibiting millions from realizing their reproductive intentions. An estimated 214 million women who want to delay or refrain from childbearing are not using contraception.^5^ As such, increasing access to voluntary family planning services is a global health goal, as demonstrated by the Sustainable Development Goals and Family Planning 2020.^6^^–^^8^ Performance-based financing (PBF) is a common approach to achieve global health goals, including family planning, because it aims to link efficient provision of high-quality health services with incentives. This approach is now one of several financial strategies of the Global Financing Facility (GFF) in support of Every Woman Every Child, the new flagship mechanism for reproductive, maternal, newborn, child, and adolescent health in 63 high-burden low- and middle-income countries^9^ (Box).

BOX **Definitions for Performance-Based Financing and Rights-Based Approach in Family Planning**

**What Is Performance-Based Financing?**

Performance-based financing is an instrument through which payments are made to providers, health facilities, or local administrative units (e.g., district health offices) for health services conditional on the performance of predefined and verified quantity indicators, adjusted for measures of quality.^10^

**What Is a Rights-Based Approach to Family Planning?**

A rights-based approach to family planning uses a set of human rights standards and principles to guide program assessment, planning, implementation, monitoring, and evaluation that enable individuals and couples to decide freely and responsibly the number and spacing of their children, to have the information and services to do so, and to be treated equitably and without discrimination.

Growing interest also exists for operationalizing rights principles in health and development initiatives to better meet client needs and ensure states fulfill their obligations to all their citizens.^11^^–^^16^ The right to health, including family planning, is universal and inalienable, and the commitment to rights-based programs implies that programs will aim for universal acceptability, accessibility, availability, and quality; accountability; agency and empowerment; equity and nondiscrimination; informed choice and decision making; participation; and privacy and confidentiality.^11^^,^^12^ Although the evidence is limited, PBF programs have the potential to strengthen person- or client-centered care with performance incentives that respect and protect individual rights. Findings are mixed, but some PBF studies have shown significant positive effects in expanding coverage, lowering consumer costs, increasing value for money, and improving the overall efficiency and quality of health systems.^17^^–^^22^ Rights-based programming—with its focus on universality—can be successfully integrated into expanded reproductive health services at minimal per capita cost, aligning with public health goals of barrier-free access and use of health care (e.g., reaching the underserved) with the promotion of universal rights.^11^^,^^13^^,^^23^^–^^25^

PBF programs could strengthen person- or client-centered care with performance incentives that respect and protect individual rights.

However, PBF program support for family planning services has often been linked to incentives for enrolling new contraceptive users or increasing clinic visits. Placing greater emphasis on person-centered needs such as client-perceived quality, counseling, or accountability could reduce the risk of discontinuation and the associated costs of unintended pregnancy while aligning with rights principles. The literature also has examples in which PBF and related results-based programs have not explicitly considered rights in their design and implementation, producing unintended negative consequences.^26^^,^^27^ Incentives paid per family planning clinic visit, for example, may unintentionally reward providers who recommend short-acting contraceptive methods, risking fully informed choice, voluntarism, and quality. Research on the implementation of PBF has shown how in practice, intended performance measures can come to be understood as targets, which have an unfortunate history in the field of family planning.^28^^,^^29^

Without a rights-based focus on clients, incentives linked to family planning services may stimulate adverse selection, with providers serving only clients who are likely to meet PBF performance standards.^28^ This gap in addressing client-centered needs, together with concerns about the risks for perverse incentives in PBF, motivated an exploration of the rights in family planning services in PBF program implementation.^11^^,^^28^^,^^30^^,^^31^

A recent literature review on performance-based financing policy draws lessons on transitioning donor-supported schemes to the health system in 4 stages (generation, adoption, institutionalization, and expansion) occurring along 5 dimensions (population coverage, service coverage, health system integration, cross-sectional diffusion, and knowledge expansion).^32^^,^^33^ This transition is defined elsewhere as the process by which a PBF project becomes an integral part of the national health system; as noted elsewhere, the process does not have a predetermined outcome.^34^ Our review of rights principles contributes to this larger discourse by suggesting another dimension to consider for PBF programs—that is, the degree to which they systematically align with a rights-based approach.

## A REVIEW OF RIGHTS IN PBF OPERATIONS MANUALS

To assess the degree of alignment between PBF programs and rights principles, we undertook an evidence mapping of PBF operational documents from November to December 2017. These operational documents were not discoverable through published literature databases. The search focused on the World Bank Group's results-based financing website, which served as a repository for PBF documentation, followed by a request to experts in the online PBF Community of Practice on Google and Collectivity groups to share additional documentation. We were unable to source all existing operational manuals because they were not available in the public domain. The exploration identified 23 relevant documents (one each from 23 countries) in English and French. The manuals were written by country teams most often with support from the World Bank, although it was not always possible to determine which donors supported the manual production. Likewise, we could not determine the level of government buy-in for specific PBF programs. Government leadership on health policy reforms, including health purchasing such as PBF, is a prerequisite for sustained success in the drive for universal health coverage.^35^ The debate regarding the degree to which PBF is a function of donor and government leadership is complex and beyond the scope of this review.^36^

We undertook an evidence mapping of PBF operational documents to assess alignment between PBF programs and rights principles.

The documents were the most recent publicly available versions of PBF operations or implementation manuals. Concepts, procedures, and performance measures were extracted and mapped to 8 rights principles for family planning sourced from global agreements, including Family Planning 2020 (2014),^12^ the World Health Organization (2014),^13^ and a revision of the Family Planning Quality of Care Framework^37^ (Table 1). The definitions and implications for each rights principle provided in Table 1 guided data extraction: any text within the PBF manuals that reflected one or more of the rights principles was selected for analysis. The evidence was extracted to a spreadsheet that tracked the degree to which each rights-based principle was included in each manual. The categories for data extraction were document type, date, each right principle, overlapping/miscellaneous indicators, and mention of family planning.

**TABLE 1.** Principles to Guide a Rights-Based Approach to Family Planning^a^

Rights Element	Family Planning Program Implications
Accessibility	Geographic, physical, financial, and policy access (i.e., absence of nonmedical eligibility criteria); information is available in the languages and terms people can understand; continuous contraceptive security; suitable hours of operation; service integration to increase access
Acceptability	Culturally appropriate facilities, methods, and services; community/family support for women's ability to choose, switch, or stop method of contraception; tolerance of side effects; privacy and confidentiality respected; client satisfaction with services
Accountability, participation, transparency	Mechanisms exist for community members and family planning clients to provide input and feedback about services, and for health system to investigate and remedy allegations of or confirmed violations of rights; members of the community are involved in planning and monitoring family planning services; good governance and effective implementation, providing an environment that facilitates the discharge of all responsibilities; and the ability to readily access meaningful information, including de-identified data.
Agency, autonomy, empowerment, voluntarism	Knowledge that one has the right to make decisions about health care; ability to make one's own decisions independent of system, husband, family, or community pressures; informed, voluntary decision making supported; meaningful participation of clients in program design and monitoring; client-controlled methods offered; supportive community gender norms; women, men, and young people know they can ask for services based on their needs, within their rights
Availability	Broad choice of methods offered; sufficient and needs-based distribution at functioning service delivery points
Informed choice, informed decision making	Women and youth and all clients make own decisions about whether and what method of family planning to use, without pressure from anyone, with free access to accurate information they can understand and a range of options to choose from
Nondiscrimination, equity	Everyone, no matter what group they come from, their age, or any other circumstance, has the same access to quality information and services; everyone is treated fairly and equitably
Quality (including privacy and confidentiality)	Service providers are well trained and provide safe services, treat clients with respect, provide good counseling, and protect client privacy and confidentiality (ensuring client information cannot be observed by anyone else without client's consent; ensuring client records are not disclosed); stock a regular supply of contraceptives and all necessary equipment to provide the services clients want

a These rights principles for family planning flow from global treaties, covenants, and conventions that define rights broadly.

Sources: Modified from Family Planning 2020 (2014),^12^ WHO (2014),^13^ and Kumar et al. (2017).^86^ Note that the definition of quality also incorporates components from the updated Bruce/Jain Quality of Care Framework for Family Planning.^37^

Data extraction was not exclusively focused on family planning–specific elements, although we gave attention to family planning implications. Identifying rights principles in the documents involved linking the terms for the rights described in Table 1 with specific elements in the operations manuals. These links were not always specific to family planning; for example, the availability of commodities is inclusive but not necessarily specific to contraceptives.

## PBF IMPLEMENTATION DOCUMENTS INCONSISTENTLY INCLUDE RIGHTS PRINCIPLES

Of the 23 implementation manuals reviewed, 21 manuals mention or include family planning. All documents focused largely on the implementation of quality and accountability mechanisms, but few addressed issues of accessibility, availability, informed choice, acceptability, and/or nondiscrimination and equity. Notably, operational inclusion of agency, autonomy, empowerment, and/or voluntarism of health care clients was absent. Table 2 illustrates which principles were included in each of the manuals.

**TABLE 2.** Rights Principles in 23 Performance-Based Financing Operational Manuals

Country	Accessibility	Acceptability	Accountability, Transparency, Participation	Agency, Autonomy, Empowerment, Voluntarism	Availability	Informed Choice, Informed Decision Making	Non-discrimination, Equity	Quality (Including Privacy and Confidentiality)	PBF Program Incentivizes FP
Tanzania	X	X	X		X	X	X	X	X
Lesotho	X	X	X		X	X		X	X
Afghanistan			X				X	X	X
Argentina	X		X				X	X	
Armenia			X						X
Benin			X		X			X	X
Burkina Faso			X						X
Burundi			X					X	X
Cameroon		X	X			X		X	X
DemocraticRepublic of the Congo			X					X	X
Djibouti			X					X	
Haiti			X					X	X
Ivory Coast			X						X
Kenya	X		X		X			X	X
Liberia			X						X
Mali			X					X	X
Mozambique	X		X				X	X	X
Nigeria			X		X		X	X	X
Rwanda		X	X					X	X
Senegal			X				X	X	X
Sierra Leone			X		X	X		X	X
Tajikistan			X				X	X	X
Zambia	X		X					X	X
**Total**	**6**	**4**	**23**	**0**	**6**	**4**	**7**	**19**	**21**

Abbreviations: FP, family planning; PBF, performance-based financing.

The manuals reviewed were produced between 2009 and 2015, prior to the GFF.^38^^–^^60^ As such, they could be updated and improved with guidance from the GFF and partner countries to include rights principles in future editions.

### Rights Are Partially Recognized in PBF Guidance

Apart from agency, all the rights principles are included across the reviewed PBF implementation documents, albeit to varying degrees. However, gaps are present in the inclusion of some principles. For example, under principles of equity and nondiscrimination, income and ethnicity were addressed by many programs, but only a few addressed youth, a critically disadvantaged and often neglected population. In addition, minimal emphasis was placed on addressing financial or information barriers to health care. For example, the operational manuals from Burkina Faso and Mozambique noted use of “equity bonus” adjustments made to PBF payments as a means to address issues of geographic access that could affect service delivery.^40^^,^^51^ Equity, as Chowdhury and colleagues note, is often cited as a PBF program indicator with its own corresponding payment mechanisms.^28^ In Mozambique, the equity bonus is paid based on the quantity of services provided in particular districts by a health facility, and it must be used for structural repairs or reproductive supply purchases. While the decision to focus on districts is one answer to resolving problems of geographic access for health care clients who are potentially more disadvantaged or vulnerable, the equity bonus does not address whether those clients are able to afford or understand available services. Burkina Faso's program provided ample detail for assessing the equity indicator. Some of the criteria used as a measure for the equity bonus include incidence of poverty in the area of service, population density, and distance between health facility and villages served.^40^ A further example of equity and nondiscrimination provided in PBF operational manuals is the payment for some services provided to the very poor in Burkina Faso and Nigeria.^52^ Nevertheless, concerns around the various monetary costs for health care paid by clients—including but not limited to travel and service costs—were not considered in most of the PBF operational manuals analyzed.

Apart from agency, all the rights principles are included across the reviewed PBF implementation documents, albeit to varying degrees.

### Client Agency Receives Limited Attention

A rights-based approach centers on clients making voluntary, informed decisions about their contraceptive use. The principle of informed choice focuses on an individual's ability to access and readily understand information about a variety of contraceptive methods and their use. This right to information is a distinct right, antecedent to agency—a right to take action, which is grouped with autonomy, empowerment, and voluntarism for purposes of this review. It implies women have the right to make decisions about having children and to act on those decisions in the health care system through knowledge of their right to family planning, voluntarily and free of discrimination, coercion, or violence. The provision of family planning options and complete information about the alternatives is one dimension of agency alongside women's capacity to articulate and drive their own decisions and to influence others to support them in achieving their goals. Aspects of agency beyond informed choice were completely absent from all the PBF manuals. Other rights principles related to agency, such as informed choice, had limited mentions in the manuals with the exception of those from Senegal and Tanzania. Informed choice focuses on providing comprehensive family planning information and a range of contraceptive options to support decision making. It is a necessary but not sufficient factor in individual agency, which entails empowering clients to actively participate in their own health care. Client agency risks being undermined by PBF incentives for the number of family planning clients served, which could result in subtle pressure or coercion to use family planning or to take a certain method. For example, a payment to a facility or provider for each family planning client served could perversely incentivize the provider to recommend short-acting methods, knowing that many clients using these methods will return for refills.^19^^,^^61^ Such an incentive could be modified to better align with a rights-based approach. For example, quality checklists and verification processes could be adapted to included validated measures of family planning clients' opportunity and confidence to ask questions during a consultation, the quality of counseling received, if they received their preferred method, and who was thought to influence their decision making.^62^^–^^65^ A recent report described what operationalizing voluntarism could look like; for instance, mechanisms could be developed to monitor for signs of coercive practices, such as setting up a confidential hotline, mobile reporting, or community dialogues.^61^

Informed choice is a necessary but not sufficient factor in individual agency, which includes clients actively participating in their own health care.

### Downward Accountability Measures Are Limited

Accountability is the only rights principle addressed in every implementation manual reviewed because it is embedded within the required verification process for PBF programs. However, the accountability measures found in the manuals only relate to internal performance checks to verify service delivery before making payments. These accountability structures connect health care providers to PBF funders and regulators but not to health care clients. Since the manuals focused on supply-side PBF programs and the contracts among funders, regulators, and health service providers, they also concentrated on these actors' respective roles and responsibilities in relation to the programs' incentives. The manuals minimally discussed how health care users should be served by the health system. In large part, the health system's responsibilities to clients, as expressed in the exit interviews, verification observations, and reviews of facility registers, focus on whether the client was served by a facility.

Previous studies of PBF have noted that verification (accountability) procedures, such as confirming poverty status or use of services, may prefer verification over the client's right to confidentiality and distorts the system's ability to monitor that client-centered services were delivered.^36^^,^^66^ In a telling example, PBF program managers in Mozambique consulted village leaders and community members as they sought to verify an HIV client's prior visit to clinic. In the process, they infringed the man's right to confidentiality, imposing significant social harm and costs on him and his household.^26^ Greater client and community participation in program design and implementation can help to align PBF accountability procedures with a rights-based approach. There are promising examples from community PBF programs in which individuals have an active role in the business plan, quality of care delivered, and identification of the beneficiaries—increasing the potential of communities providing oversight in health facilities' activities.^67^

### The Concept of Quality Is Incomplete

Quality is a common rights principle used in PBF performance measurement and incentives, as reflected in the PBF documents reviewed. Although the checklists used were structured on the accepted quality of care framework, how quality was emphasized and assessed varied across the manuals and tended to focus on tangible, structural dimensions over process indicators, such as counseling and health outcomes. Quality often overlapped with other rights principles, namely accessibility, availability, and acceptability. The wide-ranging and complex operationalization of quality remains a challenge for PBF, as for many health care programs and health systems.^68^^–^^74^ Critical elements, such as the quality of counseling beyond informed choice, received less attention, although they have been shown to have marked effects on increased contraceptive use.^75^^,^^76^

The wide-ranging and complex operationalization of quality remains a challenge for PBF.

### Concentration Is on Supply Over Demand

As is common in PBF programs, the manuals focused on supply-side financial incentives. This focus speaks to PBF design and should not be understood as a unique strategy of the GFF, which encourages countries to consider a wide range of possible approaches from high-level domestic resource mobilization, revenue pooling, and innovative financing mechanisms to improve efficiency and equity in service purchasing.^77^ Demand-side incentives were not present in the reviewed implementation manuals. The manuals often frame the PBF theory of change as an empowering, equitable, efficient, and effective approach for service providers, and a strong focus on the client is lacking. Beyond PBF, which largely focuses on supplier incentives, demand-side programs are critical to address barriers faced by clients accessing family planning services.^78^

### Limitations

This article is limited to the evidence mapping of PBF operations manuals that were available via web searches and supplemental requests to individuals. The search may have missed other PBF documents and does not include recently developed PBF manuals. Challenges in data extraction included the overlaps in definitions of rights principles. For example, accessibility and availability are necessarily related principles in obtaining family planning services. Identifying the right principle linked to an indicator such as “new contraceptive user” was challenging when the objective underlying each PBF indicator was unclear in the manual.

Another limitation is the exclusion of other kinds of results-based financing programs such as vouchers and conditional cash transfers. As noted by Musgrove,^10^ PBF is a health financing instrument that directs conditional payments to providers for health services that meet verified quantity indicators, adjusted for quality. PBF is one of multiple strategies that the GFF and the Health Results Innovation Trust Fund, which supports results-based financing to improve reproductive, maternal, and child health, have supported governments to implement. In this review, selecting only PBF manuals meant that voucher programs and other results-based financing initiatives were excluded.

## OPERATIONALIZING RIGHTS PRINCIPLES IN PBF

This review assessed current PBF design and implementation processes, inclusive of contraceptive services, against 8 rights principles.^13^ Currently, PBF manuals do not provide practical guidance on how to operationalize rights principles in a systematic way. The emphasis in the reviewed manuals is often on the autonomy of providers and facilities but not that of clients. Moreover, we noted that PBF programs are constrained by what they measure. The lack of metrics to observe certain rights would preclude alignment of PBF to rights-based frameworks. Given the significance of PBF in health care purchasing for many low- and middle-income countries, operationalizing rights principles is an urgent priority. The evolution of PBF for family planning services will have significant implications for the abilities of women and couples to choose if, when, and how many children to bear in their lifetimes, and we must ensure it is rights based, with emphasis on universal self-determination of fertility intentions.

Currently, PBF manuals do not provide practical guidance on how to operationalize rights principles in a systematic way.

A significant share of PBF technical support is administered by the World Bank, with funding from governments that have signed international agreements intended to ensure the right to the highest attainable standard of health. The GFF Business Plan states that “equity, gender, and rights underpin and are mainstreamed throughout the GFF's work,” and it provides the foundation for a new concentrated effort to explicitly integrate rights principles into the work of PBF.^79^ As the principal in providing operational guidance on the use of rights-based approaches, the World Bank can support nation-states in realizing their commitments to rights principles in PBF initiatives.

Based on this review, we recommend that this guidance include the following:

### Recommendation 1: Make the Rights-Based Approach, Centered on the Client, Explicit

Guidance on explicitly integrating a rights-based approach into PBF should start with clarifying how rights are realized within the PBF theory of change, including how such programming supports clients in addition to providers and programs. This explanation would place clients at the heart of services, ensuring they are prioritized, protected, and supported in making their own decisions about contraceptive use, within the design and implementation of PBF programs. Taking a rights-based approach would motivate the inclusion of robust indicators for quality, such as the method information index, and the development of indicators for other rights principles, such as access, choice, voluntarism, and equity. Furthermore, PBF programs built on a rights-based approach would strengthen accountability systems that move beyond financial accountability to also include community-level or social accountability for the services provided. Careful review of PBF indicators is important to ensure that the programs are not infringing on rights, for example, by denying a client informed choice by rewarding providers for provision of certain contraceptive methods over others. This articulation and prioritization of rights principles should be explicit in the guidance throughout the PBF lifecycle, from investment cases to project appraisal documents to PBF operational manuals and the monitoring and verification process, which drives the payment of incentives. The directives should be oriented first and foremost around the client, with attention to expressing quality, informed choice, voluntarism, and other rights principles in clear consistent terms throughout PBF programs. Additionally, relevant technical support should be developed to sensitize and support country teams in developing and implementing their PBF programs.

### Recommendation 2: Progressively Operationalize Rights, Drawing From Local Experience

The rights identified in the manuals serve as practical examples to be considered and taken up by other PBF programs. Current and future experiences need to be fully documented, assessed, and disseminated as a core part of PBF guidance. How rights principles are incorporated into implementation will require a thoughtful, iterative approach to document insights while accounting for contextual variation. Determining the design, implementation, and monitoring/verification of a PBF scheme as well as compensation to providers through a rights-based approach will ultimately be mediated through the local context and health system.

### Recommendation 3: Validate Rights-Based Metrics to Address Measurement Gaps

To ensure a systematic integration of rights into PBF programs, new metrics are necessary to address current gaps in measurement; in particular, metrics for agency and autonomy in the health system are needed.^80^ Secondly, the issue of a broad and complex definition of quality is a challenge. For over 25 years, the family planning field has been guided by the Bruce Quality of Care Framework.^81^ Recently, Jain proposed a revision of the framework that aligns it with definitions of quality in frameworks for rights-based family planning.^11^^–^^13^ The revised family planning quality of care framework could serve as a foundation on which quality in PBF is more clearly centered on rights.^37^

PBF programs would benefit from having a core set of indicators covering the rights principles that they could choose from to measure in their programming. This review and other recent work provide a good start at anchoring rights-based programs and aspects of PBF on metrics.^82^ New work is needed to advance a rights-based measurement agenda for family planning services in PBF and propose a list of potential indictors for validation and testing in PBF programs.

### Recommendation 4: Recognize the Economic Value of Aligning PBF With Rights Principles

The odds of successfully translating the value of a rights-based approach beyond its current community of practice may be improved by appreciating the operational enhancements that a rights-based approach could bring to PBF program performance. In this utilitarian perspective, stakeholders take into account the economic role and value of client voice and agency in PBF programs and the harm to programs that abridge those rights.^29^ If PBF services fail to meet clients' expectations for quality, confidentiality, or agency, they may impose unintentional and unaccounted costs on households. For example, if a PBF program sends field verification teams to a community where they unintentionally disclose the contraceptive use status of an adolescent PBF beneficiary, the stigma experienced by that adolescent represents a potential economic cost to them and may dissuade other young people from seeking care. Providers' negative attitudes toward adolescents' contraceptive use are well documented.^83^ Similarly, if incentivized services reward facilities for family planning patient volume instead of clients' informed choice, providers may be incentivized to disregard client choice, which places clients at risk of earlier-than-desired contraceptive discontinuation and imposes personal and social costs.^64^ Inattention to equity can result in some groups not receiving services for which they qualify.^84^ Although emerging evidence shows that higher client-perceived quality is associated with higher rates of contraceptive continuation, further research is needed to quantify the direct and indirect benefits of respectful, rights-based family planning in the context of performance-based financing.^82^^,^^85^

## Notes

**Peer Reviewed**
